# A WeChat-based self-compassion training to improve the treatment adherence of patients with schizophrenia in China: Protocol for a randomized controlled trial

**DOI:** 10.3389/fpsyg.2022.931802

**Published:** 2022-08-30

**Authors:** Die Dong, Ting-Yu Mu, Jia-Yi Xu, Jia-Ning Dai, Zhi-Nan Zhou, Qiong-Zhi Zhang, Cui-Zhen Shen

**Affiliations:** ^1^College of Nursing, Zhejiang Chinese Medical University, Hangzhou, China; ^2^Hangzhou Wenhui School, Hangzhou, China

**Keywords:** schizophrenia, treatment adherence, self-compassion, stigma, WeChat, online, research design

## Abstract

**Background:**

At present, adherence to antipsychotic treatment is often poor, leading to the recurrence of symptoms. This increases the likelihood of the patient experiencing disability and thus increases the disease burden for the patient, their family, and society as a whole. However, to date, there is no clear evidence regarding the effect of medication adherence interventions on outcomes for patients with schizophrenia. Moreover, the traditional intervention methods are limited by manpower and resources in low- and middle-income countries. Recent studies have demonstrated that increasing a patient’s level of self-compassion may improve their treatment adherence. Online mental health care interventions have advantages in terms of feasibility and acceptability for patients with schizophrenia. In this regard, a WeChat-based self-compassion training protocol to improve patient treatment adherence was designed in this study and will be evaluated in the future to determine its impact on patients with schizophrenia.

**Methods:**

The protocol for the randomized controlled trial (RCT) is based on the SPIRIT 2013 statement. This parallel RCT will aim to recruit 392 patients with schizophrenia who will be randomized at a 1:1 ratio into a 3-week intervention or control group. Both groups will receive routine care. The intervention group will also receive WeChat-based self-compassion training, which requires participants to complete three tasks every day, including a reading task, a meditation task, and a self-compassion journal task. The control group will receive WeChat-based psychological health education, which will only require participants to read positive articles about psychological health every day. Medication adherence, self-compassion, stigma, and social support will be measured at baseline (T_0_), immediately after the intervention (T_1_), and 3 weeks after the intervention (T_2_). Program feasibility will be evaluated throughout the course of the study, and acceptability will be measured immediately after the intervention (T_1_).

**Expected results:**

The intervention described here will address the barriers to accessing mental health care for people with schizophrenia, including patients’ desire for independent management, difficulty accessing providers, and concerns about privacy and stigma. The current study provides guidance for clinical nurses to carry out psychological intervention, with the ultimate aim of addressing the problems associated with a shortage of psychological professionals in low- and middle-income countries.

## Introduction

Most guidelines recommend continuous treatment with antipsychotic medication to prevent relapse or recurrence of psychotic symptoms during the first few years following the first psychotic episode (Galletly et al., 2016; Crockford and Addington, 2017; Shimomura et al., 2020). However, studies have shown that adherence to antipsychotic treatment is often poor; between 41 and 61% of patients do not take the medication as prescribed (Valenstein et al., 2006), and the reported relapse rate is 42% due to poor medication compliance in patients with chronic schizophrenia (Acosta et al., 2009). In China, 55∼76% of patients with schizophrenia relapse because of drug withdrawal (He and Li, 2018). High recurrence not only increases the possibility of disability but also results in a serious disease burden for the families of patients and society. According to previous reports, the disability rate in schizophrenia is as high as 92.50% (Insel, 2010). Inclusion of disability when measuring disease burden has been particularly influential in highlighting schizophrenia as a leading contributor to disease burden. Observing differences in disability-adjusted life years (DALYs) demonstrates that the large burden of schizophrenia experienced in lower- and middle-income countries is around four times the burden experienced by high-income countries (Charlson et al., 2018). This is largely attributable to the significant population growth and aging of low- and middle-income countries, resulting in a greater proportion of the population of an age where the risk of schizophrenia is greatest (Charlson et al., 2018). Therefore, services to improve treatment adherence and prevent disease recurrence are important to respond to the high disability rate and disease burden among patients with schizophrenia, particularly in low- and middle-income countries.

Uzer-Kremers et al. (2020) first explored the relationship between self-compassion and treatment adherence among patients with schizophrenia in an investigation study. The results indicated that improving the self-compassion of patients with schizophrenia may increase their level of treatment adherence. Mindfulness-based interventions (Rimes and Wingrove, 2011), compassion-focused therapy (CFT) (Gilbert, 2014), and mindful self-compassion (MSC) (Neff and Germer, 2013) are training programs and intervention techniques proposed to increase a patient’s level of self-compassion. However, most of these therapies are based on group interventions, which are complex and require the input of professionals. Low- and middle-income countries lack the mental health professionals required for such interventions (McKenzie et al., 2004). Moreover, given the widespread public discrimination and prejudice against mental illness, seeking face-to-face psychotherapy is often challenging for patients with schizophrenia. With the development of the Internet, several researchers have conducted online interventions, which have advantages in terms of feasibility and acceptability and may lower the treatment-seeking threshold for patients (Josephine et al., 2017). The published literature to date also provides strong evidence for the feasibility of using smartphones to enhance the treatment adherence of people with schizophrenia (Firth and Torous, 2015). In recent years, online interventions based on self-compassion for patients with mental illness have been developed and have demonstrated positive effects. However, to date, there are no published studies to support the effectiveness of online self-compassion training among patients with schizophrenia.

## Background

While some studies of existing psychological interventions have demonstrated their effectiveness in improving medication adherence among patients with schizophrenia, including compliance therapy (CT), adherence therapy (AT), cognitive behavior therapy (CBT), and motivational interviewing (MI), other studies have reported that these interventions do not improve medication adherence (O’Donnell et al., 2003; Bechdolf et al., 2005; Barkhof et al., 2013; Schulz et al., 2013). The CT or AT or MI is multicomponent and complex, differs widely in their content and implementation, and lacks standardized interventions for patients with schizophrenia, leading to different research results (Hartung et al., 2017). Furthermore, people who are highly self-critical and shame-centered tend to respond poorly to standard CBT and consider CBT to be “emotionally heavy and unpleasant” (Barnes et al., 2013). A systematic review found no clear evidence to support the effectiveness of these medication adherence interventions on the outcomes of patients with schizophrenia (Hartung et al., 2017). Nonetheless, interventions to improve medication adherence among individuals with psychotic symptoms warrant further investigation (Hartung et al., 2017). Moreover, many of the interventions currently available require significant manpower and resources, making them less practical for low- and middle-income countries that lack these mental health professionals (McKenzie et al., 2004).

Recent theoretical developments suggest that self-to-self relating may be a critical process in the recovery of psychosis (Andrew et al., 2010). Self-to-self relating refers to the way in which individuals relate to themselves; it can be considered as an intrapersonal relationship (Andrew et al., 2010). Self-criticism causes distressing experiences of psychosis, whereas compassionate self-acceptance results in empowered action and promotes recovery and growth during psychiatric rehabilitation (Waite et al., 2015). However, during rehabilitation, patients with psychosis often adopt self-criticism to deal with their difficulties and maintain their self-esteem and self-identification (Lawrence and Lee, 2014). Highly self-critical people habitually experience feelings of inferiority, worthlessness, shame, failure, and guilt, and are often reluctant to seek support (Krieger et al., 2019). The higher the level of self-criticism, the more shame people will experience (Krieger et al., 2016). Furthermore, one-third to half of all patients with schizophrenia feel ashamed for suffering from this disease, and the stigma persists in the remission period of the disease (Gerlinger et al., 2013). Patients will select strategies such as confidentiality or isolation rather than help-seeking to avoid these feelings (Cadario et al., 2012). Stigma affects all stages of treatment, from attitude and approach to treatment plan selection (Stentzel et al., 2018). In clinical work, due to the fear of drug side effects and medicine-taking behavior that may expose their disease, patients with schizophrenia will reduce or even stop the drug themselves to avoid stigma (He, 2018).

The most common intervention that focuses on self-criticism and the stigma of mental illness is cognitive behavioral therapy (CBT), which is to challenge the content of negative thoughts, address cognitive biases in the processing of emotional information, and alleviate psychological symptoms (Vazquez et al., 2018). However, studies have shown that the influence of CBT may be overestimated (Cuijpers et al., 2016). Further, people who are highly self-critical and shame-centered tend to respond poorly to standard CBT and consider CBT to be “emotionally heavy and unpleasant” (Barnes et al., 2013). Self-compassion is a positive emotion regulation strategy and emotional arousal state (Neff and Germer, 2013). People with high levels of self-compassion tend to respond with understanding and acceptance when facing difficult emotions, rather than with avoidance (Neff and Germer, 2013). Some qualitative studies have also demonstrated the effect of self-compassion on patients with mental illnesses (Ashworth et al., 2015; Ashfield et al., 2020; Bratt et al., 2020). After completing self-compassion training, patients tend to accept rather than criticize themselves as having a problem. In recent years, scholars have demonstrated the value of self-compassion for treatment adherence in a variety of clinical populations, including patients with fibromyalgia, chronic fatigue syndrome, and cancer (Sirois and Hirsch, 2019). The relationship between self-compassion and treatment adherence among patients with schizophrenia has also been explored in an investigation study, with the results suggesting that improving self-compassion may increase the treatment adherence of patients with schizophrenia (Uzer-Kremers et al., 2020). Specifically, self-compassion supports individuals in developing a compassionate attitude toward themselves, prompting them to accept and understand their own diseases, thus reducing self-criticism and stigma and assisting in the maintenance of a positive attitude toward their responsibility for their own treatment.

There are many training programs and intervention techniques proposed to increase a patient’s level of self-compassion, including mindfulness-based interventions, MSC, and CFT. CFT was developed specifically to build the capacity to experience compassion in high-shame and self-critical individuals (Gilbert, 2014); Braehler et al. (2013) randomized patients with a schizophrenia-spectrum disorder to either a CFT group or a control group. During the 4-month CFT, compassion skills, such as mindfulness, appreciation, imagery, attention, behavior, and reframing, were practiced, and expressive writing tasks were used to help members reflect on and integrate changes in their recovery from a compassionate stance. Finally, the results support the feasibility of group CFT in psychosis and suggest that changes in compassion can be achieved.

In recent years, new forms of online interventions based on self-compassion meditation and self-compassion writing therapy for patients with mental illness have been developed and have demonstrated positive effects. The intervention time is also getting shorter [6 weeks (Finlay-Jones et al., 2017; Rodgers et al., 2018; Andersson et al., 2020), 4 weeks (Mak et al., 2018, 2019; Beshai et al., 2020), 3 weeks (Albertson et al., 2015; Toole and Craighead, 2016), 2 weeks (Kelman et al., 2018; Stevenson et al., 2019; Halamová et al., 2020, 2021; Schnepper et al., 2020; Seekis et al., 2020), and 1 week (Shapira and Mongrain, 2010) or less (Galla, 2016)]. For example, Albertson et al. (2015) randomized participants to either an intervention group (meditation via podcast) or a waitlist control group. The results indicated that the participants in the intervention group experienced significantly greater reductions in body dissatisfaction and body shame and greater gains in self-compassion and body appreciation after the 3-week meditation intervention, when compared to the control group. However, to date, there are no published studies to support the effectiveness of online self-compassion training among patients with schizophrenia. Compared with group interventions in a therapy room, online interventions can address barriers to accessing mental health care, such as patients’ desire for independent management, limited funds, time constraints, transportation issues, difficulty accessing providers, and concerns about privacy and stigma (Josephine et al., 2017). Online interventions can also reduce the use of mental health resources in low- and middle-income countries, where these resources are scarce. A recent meta-analysis demonstrated that online interventions are as effective as face-to-face interventions for a variety of psychological and physical diseases (Carlbring et al., 2018). For example, the FOCUS smartphone app is a multifaceted mobile intervention that targets auditory hallucinations, mood, sleep, functioning, and medication adherence (Ben-Zeev et al., 2013). FOCUS has demonstrated similar efficacy to standard in-person psychosocial approaches in caring for people with schizophrenia. Other smartphone apps have also been effectively designed to address social functioning in patients with schizophrenia (Fulford et al., 2021). Therefore, it is meaningful to design an online self-compassion training program specifically for patients with schizophrenia.

The existing research on self-compassion has mostly been conducted in English-speaking countries. However, self-compassion theory is, in fact, inspired by Eastern Buddhism. Studying the localization of self-compassion in China is of great practical significance, and the development of an online intervention is in line with the inadequate mental health resources in China (McKenzie et al., 2004). WeChat is a free smartphone application; it is the most widely used social networking platform in China. Similar to Facebook, WeChat public accounts can send push notifications to their followers and alert them to new content. WeChat can be used to deliver health education messages in a cheaper and more visually appealing way compared to mobile phone SMS. Studies conducted in China have shown that WeChat is effective in health promotion interventions (He et al., 2017).

Considering the importance of improving treatment adherence, the effectiveness of a high level of self-compassion, the advantages of online interventions for patients with schizophrenia, and the widespread use of WeChat among people in China, this study describes a brief WeChat-based self-compassion training protocol to improve the treatment adherence of patients with schizophrenia in China.

### Theoretical framework

The intervention program described in this study is based on four principles. First, self-compassion theory was taken as the theoretical basis. Self-compassion comprises three interacting components: self-kindness vs. self-criticism, a sense of common humanity vs. isolation, and mindfulness vs. over-identification (Neff and Germer, 2013). Self-kindness refers to the tendency to be caring and understanding toward the self rather than being harshly judgmental. Rather than attacking and berating oneself for personal shortcomings, the self is offered warmth, comfort, and unconditional acceptance. The sense of common humanity that comprises self-compassion involves recognizing that all people are imperfect, fail, make mistakes, and experience serious life challenges, rather than feeling isolated by the experience of imperfection. Mindfulness in the context of self-compassion involves being aware of one’s painful experiences in a balanced way, which neither ignores nor amplifies painful thoughts and emotions. In addition, paying attention in an equilibrated way is important; this does not involve “over-identification,” that is, being carried away by a dramatic storyline that exaggerates implications for self-worth. Therefore, we believe that patients with a high level of self-compassion tend to be caring and understanding rather than harshly judgmental about their diseases. They recognize that everyone will experience serious life challenges and be aware of their painful experiences in a balanced way, thus reducing self-criticism and stigma, and maintaining a positive attitude toward their responsibility for their own treatment.

First, the online self-compassion intervention studies in the literature were reviewed to determine the intervention content and intervention dose, including intervention form, frequency, time, and duration. The intervention aims to help participants to understand the meaning of self-compassion and to use the three components of self-compassion to help them actively cope with self-criticism and stigma, thereby improving their treatment adherence. Second, structured interviews were conducted with the target population (patients and nurses in an inpatient ward) to ensure that the program met the health needs of patients with schizophrenia and was consistent with the actual clinical situation. Third, an expert meeting (a group of eight experts including two senior doctors and two nurses in the field of schizophrenia, two psychologists, and two nursing experts) was held to ensure the scientific basis and feasibility of the intervention program. Finally, a research group meeting was conducted to identify all the detailed messages that would be published on the WeChat public account.

## Study

### Aim

The ultimate goal of this research is to evaluate the effectiveness, feasibility, and acceptability of a WeChat-based self-compassion training protocol to improve the treatment adherence of patients with schizophrenia in China. The specific objectives of the study are as follows:

#### Primary aim

To evaluate the effects of the WeChat-based self-compassion training on treatment adherence, relative to the control WeChat-based psychological health education.

#### Secondary aim

To evaluate the effects of the WeChat-based self-compassion training program on self-compassion, stigma, and social support; evaluate the feasibility of online training by examining enrollment, recruitment, retention, and journal task completion rates; and determine satisfaction and acceptability of the program by assessing eight single items on the intervention satisfaction scale.

### Design

#### Trial design

**FIGURE 1:**
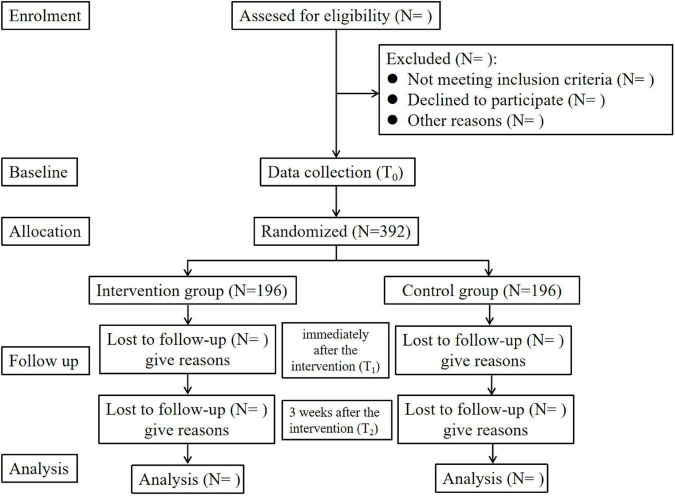
An overview of the study design.

The study will be an exploratory, parallel randomized controlled trial (RCT) comparing a 3-week WeChat-based self-compassion training protocol to a control condition with only WeChat-based psychological health education. The effects of the intervention on treatment adherence, self-compassion, stigma, and social support will be evaluated among patients with schizophrenia. Eligible participants will be randomly allocated to one of the two conditions (intervention or control) at a ratio of 1:1. The Consolidated Standards of Reporting Trials (CONSORT) (Schulz et al., 2010) flowchart is presented in Figure 1. The study protocol was developed and reported according to the Standard Protocol Items: Recommendations for Interventional Trials (SPIRIT) 2013 statement (Chan et al., 2013; Supplementary File 1).

#### Study setting

Participants will be recruited from the psychiatric wards at two hospitals in Hangzhou, Zhejiang, China. Both hospitals are tertiary public hospitals covering almost all patients with schizophrenia who seek medical help in Hangzhou. There is little difference in the clinical education and treatment protocols between the two hospitals. The intervention will be performed online.

#### Eligibility criteria

The research coordinator will confirm the eligibility criteria for each participant by screening their electronic medical records and communicating with the patient and their family members face-to-face.

**Inclusion criteria:**

(1) Patient must have been diagnosed with schizophrenia according to the International Classification of Diseases (ICD-10) criteria for the past year.
(2) Patients must be over 18 and under 65 years.
(3) Patient has achieved a curative effect that meets the clinical recovery standard according to the evaluation of psychiatrists.
(4) Patient must have normal cognitive, expression, and comprehension abilities and the ability to fill in the questionnaire correctly.
(5) Patient must own a smartphone and be able to use WeChat.
(6) Informed consent must be obtained from the patient and one of their family members.

**Exclusion criteria:**

(1) ] Patients suffering from other mental disorders.
(2) Patients with severe organic brain lesions and physical diseases.
(3) Patients participating in other psychological interventions.
(4) Patients who have previously received clinical guidance similar to that provided in this study.

**Rejection criteria:**

The rejection criteria are as follows: patients who did not meet the inclusion criteria and who met the exclusion criteria but were mistakenly recruited into the study.

**Abscission criteria:**

(1) Patients have deteriorated mental conditions during the course of the study.
(2) Patients quit the study.
(3) Patients could not be contacted during the intervention, and they did not complete the intervention tasks as required.
(4) Patients did not complete post-intervention evaluation.

#### Sample size

The sample size of the study was calculated using the G*Power 3.1 software package (Faul et al., 2007). With a power of 0.80, an alpha set at 0.05, and an effect size of 0.3 for the primary outcome, the Medication Adherence Rating Scale (MARS) score (Çetin and Aylaz, 2018), it was determined that each group would need 176 patients with schizophrenia. Given an attrition rate of 10%, a minimum of 392 participants (196 in each group) is required.

#### Recruitment, randomization, and allocation

Potential participants in the hospital will be identified by screening the electronic medical records on each psychiatric ward to determine those who have achieved a curative effect that meets the clinical recovery standard. These potential participants will then be contacted by a research coordinator to determine their eligibility, their ability to fill in the questionnaire, their interest in participation, their experience using WeChat, and their willingness to sign the consent form (including one of their family members). The aims of the study and participants’ right to withdraw at any time will be explained to eligible participants verbally and via an information sheet. Individuals will be able to ask the research coordinator questions about the study.

After signing the written informed consent and completing the basic demographic questionnaire and baseline evaluation, all eligible participants will be randomized to either the intervention group or the control group at a 1:1 ratio. Participants will be randomized individually using a computer-generated sequence of random numbers generated with SPSS 25. Allocation concealment will be achieved through sequentially numbered, opaque, sealed envelopes. The random sequence will be generated by an independent researcher not involved in patient enrollment and the baseline assessment. The envelope will only be opened after obtaining consent, confirming the patient’s eligibility, and performing the baseline evaluation. Then, the responsible clinical psychiatric nurses, who will facilitate the intervention in this study, will be informed of the intervention allocation. Given that the participants will either receive direct access to the WeChat-based self-compassion training or the WeChat-based psychological health education, participants will be blind to their allocation.

Participants assigned to the WeChat-based self-compassion training will receive access to the intervention WeChat group. The control group will receive access to another WeChat group. The two WeChat groups will be set with management authority such that the patients will not be able to enter the group without the consent of the responsible clinical psychiatric nurses, who will be the managers of the WeChat groups. All participants in the intervention WeChat group will be asked to scan a two-dimensional code to subscribe to and register with the “Self-compassion Tour” module of the WeChat public account. All participants in the control WeChat group will also be asked to scan another two-dimensional code to follow a WeChat public account in order to read positive articles about psychological health. Because they will not be able to register with the “Self-compassion Tour” module, they will not be able to access the information in the module. This will avoid between-group contamination through the direct sharing of messages sent via WeChat.

#### Intervention

**FIGURE 2:**
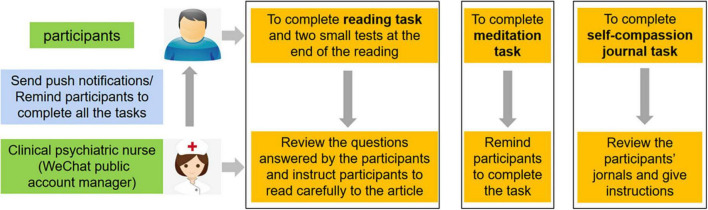
Function of the ‘Self-compassion Tour’ system. Figure created with Microsoft Office PowerPoint.

**FIGURE 3:**
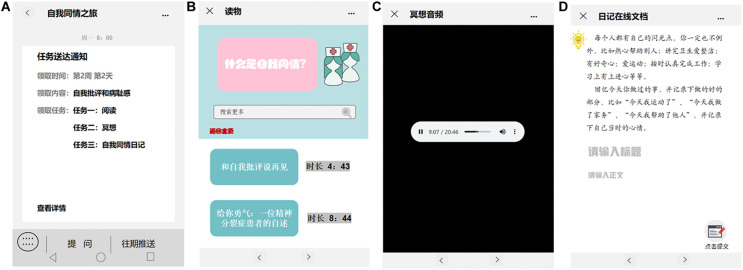
The interface of the ‘Self-compassion Tour’. **(A)** Notification of new messages, **(B)** reading task, **(C)** meditation task, **(D)** self-compassion journal task. Figure created with Microsoft Office PowerPoint.

The clinical psychiatric nurses will run the intervention. They will be trained face-to-face for 2 days by the research team and will be required to master the contents and methods of the WeChat-based self-compassion intervention. Clinical psychiatric nurses who pass the examination will act as the nurses responsible for the WeChat-based self-compassion training intervention. The training protocol developed by the researcher is totally independent and different from the clinical education received at the hospital. The “Self-compassion Tour” module includes the following three tasks: a reading task, a meditation task, and a self-compassion journal task. Each message in the intervention will be compiled by one research team member who will develop appropriate multimedia content using videos, pictures, or records to effectively convey the meaning of each message. Each message will be revised according to expert opinion and subsequently approved by the research team members. The messages will be published on the WeChat public account “Self-compassion Tour” every day on time. Each task is described in detail below. The key themes of the “Self-compassion Tour” are summarized in Table 1. The function of the “Self-compassion Tour” system is shown in Figure 2. The interface of the “Self-compassion Tour” is shown in Figure 3.

**TABLE 1:** Key themes of the “Self-compassion Tour”.

Week	Theme	Task		
		
		Reading task	Meditation task	Self-compassion journal task
Week 1	Understanding self-compassion	Introduction of self-compassion	The compassionate body scan	Keep a journal from perspectives of mindfulness, a sense of common humanity and kindness
Week 2	Understanding self-criticism and stigma	Stories of being self-kindness to oneself who are also suffering from schizophrenia	loving-kindness meditation	Keep a journal from perspectives of finding your own strengths
Week 3	Improve treatment adherence	Stories of Coping with difficult feelings and emotions of adhering to treatment	Affectionate Breathing	Keep a journal from perspectives of encouraging yourself to recovery

##### Reading task

The content provided for reading will be updated daily according to the theme of the week. Participants will be able to read the new messages and review the message history of all content published on the WeChat public account. The messages will be shown in the form of text, videos, and pictures. The content of the first week will be focused on theoretical knowledge in relation to self-compassion. The aim is for participants to understand the meaning of the three levels of self-compassion and be able to start the self-compassion journal task. The second and third weeks will be focused on positive stories about people suffering from schizophrenia. These 2 weeks are a complement to the “sense of common humanity” gained in group therapy, where patients are able to view their experiences as part of a common experience of many people with schizophrenia (Lawrence and Lee, 2014), fostering acceptance by other group members (Ashfield et al., 2020). Reading new messages on the WeChat group will take approximately 10 min every day. At the end of the reading, two small tests will be inserted to check whether the participant has read the content carefully.

##### Meditation task

In the 3-week online self-compassion meditation intervention, participants will be asked to listen to an audio recording regularly each week (at least once per day) (Albertson et al., 2015). Three different guided self-compassion meditations that were taught in the MSC program will be used for the intervention (Neff and Germer, 2013). For example, the first week will be the “compassionate body scan,” which aims to help the participant feel connected with their body and give their body sympathy, peace, and gratitude. Accompanied by the audio, the participants will be asked to sit or lie down, put their hands on their heart, remind themselves to be kind to themselves, and then scan from the top of their head to their feet and pay attention to the feeling of every part of the body. The task will take approximately 10 min every day.

##### Self-compassion journal task

This task is a modification of the self-compassion journal exercise on Professor Neff’s self-compassion website.^1^ The content of the weekly journal will differ in relation to the theme of the week. For example, in the first week, participants will be asked to “keep a journal from the perspective of mindfulness, a sense of common humanity and kindness.” They will be presented with prompts, such as “at some point during the day when you have a few quiet moments, write in your journal about anything that you felt bad about, anything you judged yourself for, or any difficult experience that caused you pain. For each event, use mindfulness, a sense of common humanity, and kindness to process the event in a more self-compassionate way.” Participants will be asked to try to write in their daily self-compassion journal for approximately 15 min, with guidance. All diaries will be delivered to the clinical psychiatric nurses via online documentation links on the WeChat public account, and participants will be instructed on the basis of their writing in order to better use the three components of self-compassion to help them actively cope with difficult emotions.

Task completion will be tracked every day. When the participant completes the task for the day, they will be required to click “I have completed the task” in the WeChat group; if the participant fails to complete the task before 8.00 p.m. that day, they will receive a private WeChat reminder message from the clinical psychiatric nurse. If participants have any questions about task completion, they will be able to leave a message on the WeChat public account, and the clinical psychiatric nurse will reply as soon as possible. All questions and answers on the WeChat public account will be visible to all participants in the intervention group (all the participants will be assured of anonymity by using special code numbers to identify themselves). The information forwarding function will be disabled, and participants will be told not to share the messages with others, such as by taking screenshots, in order to minimize potential contamination.

The control group will follow another WeChat public account which will provide psychological health education. Participants in the control group will only be required to read excerpts of articles from books about psychological health. The articles will be organized into three sections (one section per week): challenges faced by those with mental disorders, signs of bad emotions, and psychological health self-management recommendations. The articles will be updated daily for 3 consecutive weeks.

Adverse events, if any, are expected to be minor. At the start of every week, participants will be asked whether they experienced any physical discomfort, emotional discomfort or distress, or an increase in problems in relationships with others in the past week. With regard to reporting adverse events, data from the intervention and control arms will be combined, and descriptive statistics will be used to evaluate the frequency and severity of adverse events. All collected adverse events will be reported.

#### Instruments and measures

##### Demographic and other personal data

Participants’ demographic information will be collected, including gender, age, previous residence, marital status, education, economic status, employment status, and duration of illness.

#### Primary outcomes

##### Patient adherence

The Medication Adherence Rating Scale (MARS) (Thompson et al., 2000) will be used in our study. The 10-item measure assesses patients’ attitudes, beliefs, and behavior toward taking medicine on a scale set to “yes” and “no” options for each item. The MARS is a reliable tool to measure medication compliance of patients with schizophrenia 1 week before the measurement point (Friemann and Wciórka, 2013).

#### Secondary outcomes

##### Self-compassion

Self-compassion will be measured using the Self-Compassion Scale (SCS) (Neff, 2003). This self-reported scale consists of 26 items designed to measure the six subcomponents of self-compassion: mindfulness, over-identification, sense of common humanity, isolation, self-kindness, and self-judgment; each item is rated on a 5-point scale. Item scores are used to generate a total self-compassion score. In the present study, the Chinese version of the SCS has shown good values for reliability and validity, and Cronbach’s α is set at 0.84 for the total score (Chen et al., 2011).

##### Stigma

Stigma will be measured using the Stigma Assessment Scale for mental illness, particularly in Chinese, a widely used measure of stigma (Zeng et al., 2009). This self-reported scale measures stigma along three dimensions: (1) socialization (related to the discrimination of patients in social and interpersonal communication), (2) competence (involves patients experiencing public discrimination against their personal abilities), and (3) therapy (related to patients’ experience of treatment, side effects, and neglect by medical staff). The scale is a self-reported measure comprising 32 items, and each item is rated on a 4-point scale. Item scores are used to generate a total stigma score. The scale has demonstrated good internal consistency in mental illness samples, and Cronbach’s α is set at 0.9.

##### Social support

Social support will be measured using the Social Support Rating Scale (SSRS) (Xiao and Yang, 1987). This self-reported scale consists of 10 items designed to measure the three dimensions of an individual’s social support: objective support (the actual support received by the patient), subjective support (the support that the patient can experience or emotional support), and support utilization (the active use of various social supports by individuals, including the way of talking, the way of asking for help, and the way of participating in activities). Item scores are used to generate a total social support score, and the higher the total score, the better the degree of social support. SSRS has good reliability and validity with Cronbach α and KMO of 0.72 and 0.637, respectively.

##### Program feasibility

Program enrollment, recruitment, retention, and diary completion data will be gathered to measure program feasibility: (1) enrollment rate = number of patients who meet inclusion criteria/number of people with schizophrenia; (2) recruitment rate = number of patients who agreed to participate in the study/number of patients who meet the inclusion criteria; (3) retention rate = number of patients who complete the intervention/number of patients who agreed to participate in the study; and (4) diary completion rate = actual number of completed journals/required number of completed journals.

##### Program acceptability

Participants’ satisfaction with and acceptability of the intervention will be assessed immediately after the intervention using eight single items on the Intervention Satisfaction Scale, which refers to the Intervention Satisfaction Scale by Campo et al. (2017). The content includes the acceptability of the intervention (two items) and the satisfaction of the intervention plan and implementation (six items), such as the frequency, duration, and length of the training. Our primary benchmark for acceptability is an average score of 4 or higher on participants’ responses to two items on this scale: (1) “Overall, I really enjoyed the self-compassion training” and (2) “I would recommend the self-compassion training to other patients with schizophrenia.” The other six items assessing satisfaction with the intervention are set to “yes” and “no” options for each item: “Generally speaking, I prefer online training,” “For me, a total of 3 weeks of training is appropriate,” “For me, about 45 min a day for training is the right length,” “For me, once a day of every task for 3 weeks is the right frequency,” “I am satisfied with the content of the training,” and “For me, most of the time I can complete my training in a quiet and undisturbed environment.”

#### Data collection and management

Table 2 shows the schedule of enrollment, interventions, and assessments. Feasibility outcomes will be evaluated throughout the course of the study. Other data will be collected online using questionnaires presented in an online format.^2^ Measurements will be taken at baseline (T_0_), immediately after the intervention (T_1_), and 3 weeks after the intervention (T_2_). Participants will complete the baseline measurement by scanning a two-dimensional code to obtain their questionnaire. At T_1_ and T_2_, the questionnaires will be sent to participants by WeChat. All participants will receive up to two reminders to complete the T_1_ and T_2_ measurements, regardless of protocol adherence or any previously uncompleted online questionnaires. Participants will be informed that they will receive a gift after completing the online questionnaires to promote retention.

**TABLE 2:** SPIRIT figure, schedule of enrollment, interventions, and assessments.

	Study period				
	
	Enrollment	Allocation	Post-allocation		
			
			Intervention		

Timepoint	T_0_	0	0	T_1_	T_2_
**Enrollment:**					
Eligibility screen	×				
Informed consent	×				
Allocation		×			
**Intervention**					
Intervention group (WeChat-based self-compassion training)		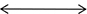			
Control group (WeChat-based psychological health education)		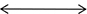			
**Instruments**					
Demographic and other personal data	×				
**Primary outcomes**					
Patient adherence	×			×	×
**Secondary outcomes**					
Self-compassion	×			×	×
Stigma	×			×	×
Social suppor	×			×	×
Program feasibility		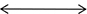		×	
Program acceptability				×	

Online website servers will be used to collect all data, according to the hospital security guidelines and policies; all web-based information transmission will be encrypted. Baseline data and follow-up data will be saved first on the website and then wirelessly uploaded into an Excel database via an Internet server. Data entry will be accomplished by individuals external to the research team, and data analysis will be completed without referring to the allocation information.

#### Statistical methods

The baseline characteristics of the sample will be compared using χ^2^-tests for categorical variables and Student’s *t*-test or Mann–Whitney *U*-test for quantitative variables.

Statistical analyses will be performed on the basis of the intention-to-treat approach, which will include all randomized participants. The extent of missing data will be analyzed. We will explore missing data patterns and determine the type of missing data (missing completely at random, missing at random, and not missing at random). We will use multiple imputations to substitute missing values and conduct sensitivity analyses for datasets with and without imputed data.

Descriptive statistics will be used to evaluate study feasibility and acceptability. We will use linear mixed models with time (pre-intervention vs. post-intervention measures) as a within-group effect, study condition (intervention condition vs. control condition) as a between-group effect, and the interaction effect between these two effects to evaluate the efficacy of the WeChat-based self-compassion training intervention on patient adherence, self-compassion, stigma, and social support. This primary analysis will be performed using the data from the baseline and 3-week post-assessment. We will conduct within-group analyses using repeated measures ANOVA (pre-intervention, post-intervention, and follow-up measures) and paired *t*-tests when comparing only two time points to analyze the stability of the short-term effects of the intervention on patient adherence, self-compassion, stigma, and social support. Moreover, the correlation among self-compassion, stigma, social support, and patient adherence will be analyzed using Pearson correlation. The results will be reported with 95% confidence intervals. Bilateral *p* ≤ 0.05 shows a statistically significant difference. All data will be analyzed using SPSS 24.0.

### Ethical considerations

This study was approved by the Human Research Ethics Committee (REDACTED). All participants and one of their family members will be required to sign an informed consent form, which includes details of the intervention goals and procedures. Interventions and questionnaires will be conducted after written informed consent is obtained from each recruited participant. Participants will be informed of the freedom to withdraw from the study at any time and will be assured of anonymity by using special code numbers to identify themselves. All of the collected data will be pseudo-anonymized and kept confidential. Only members of the research team will be able to re-identify the participants. If the intervention proves to be effective, then participants in the control group will also receive the WeChat-based self-compassion training after the study is completed. The trial is registered at the Chinese Clinical Trials.gov.

### Validity and reliability

This study uses a rigorous research design, an RCT with a representative and predetermined sample. It uses instruments with high validity, reliability, and statistical analysis, which can effectively reduce bias and enhance the generalizability of research results beyond the target population. Moreover, trial participants, outcome assessors (no researchers will be directly involved during data collection, as questionnaires are online), and data analysts of the research will be blinded to intervention allocation to reduce the biases in the evaluation of the effects of the intervention. The study protocol is in accordance with the SPIRIT reporting guidelines (Chan et al., 2013).

## Discussion

Although previous research supports the notion that psychological outcomes among people living with mental illness can be improved following online-based self-compassion interventions, to date, there are no published studies on this issue among patients with schizophrenia. We expect that the psychological program described here will help patients with schizophrenia to increase their level of self-compassion, cope with experiences of self-criticism and stigma, and avoid confidentiality and unsociability, so as to ultimately improve treatment adherence. The planned RCT will contribute evidence on the effectiveness of using the WeChat platform to support patients with schizophrenia. Online training is a flexible, low-cost, sustainable mode of delivery. It also addresses the barriers to accessing mental health care for people with schizophrenia, including patients’ desire for independent management, difficulty accessing providers, and concerns about privacy and stigma. This study provides guidance for clinical nurses to carry out psychological intervention so as to improve treatment adherence among patients with schizophrenia. The evidence obtained from the described RCT, which will be led by clinical nurses, will help to address the problem of a shortage of psychological professionals in low- and middle-income countries and will also provide information for the provision of mental health services and policies. Furthermore, the self-compassion knowledge gained from this study could be used to plan a culturally appropriate program for people with other mental illnesses. This could be particularly useful during the COVID global pandemic when access to mental health services may be limited due to restricted movement, and web-based options may be more accessible.

### Limitations

Although we have carefully crafted this protocol, this study has some limitations. First, this intervention will be conducted for 3 weeks, and some participants may not be able to complete the study because of changes in their emotions or loss of follow-up. Second, we may not be able to avoid participation bias, as participation in the study is voluntary. Third, restricting the study to patients with a smartphone and being able to use it may cause a selection bias, which could reduce the generalizability of our results. This limitation is common in online intervention. Finally, we will not be able to monitor the level of usage of the WeChat public accounts during the trial because of the limited function of the WeChat public account; therefore, determining the extent to which higher intervention adherence is associated with higher benefits on outcomes will not be possible.

## Conclusion

In this study, the scientific problem was first identified by reading the current literature. Specifically, it is clear that patients with schizophrenia often adopt self-criticism and feel shame when dealing with their difficulties, leading to poor medication adherence. Then, the research group established a conceptual framework around this problem, specifically self-compassion theory and the relationship between medication adherence and self-compassion. Then, an evidence-based intervention program was established through literature review; structured interviews with patients and nursing staff were conducted to modify the intervention program from the perspective of the stakeholders. Finally, the intervention program was professionally perfected through expert meetings. In the next step, our research group will formally implement this RCT. If this online intervention for patients with schizophrenia is effective, it will then be implemented in hospitals and communities to improve treatment adherence among patients with schizophrenia in China.

## Data availability statement

The original contributions presented in this study are included in the article/Supplementary material, further inquiries can be directed to the corresponding author.

## Ethics statement

The studies involving human participants were reviewed and approved by the Medical Ethics Committee of Zhejiang Chinese Medical University. The patients/participants provided their written informed consent to participate in this study.

## Author contributions

DD and C-ZS conceived of the study. T-YM, J-YX, Z-NZ, J-ND, and Q-ZZ collected material. DD drafted the manuscript. DD and T-YM met with C-ZS to discuss the protocol. All authors helped to draft and approved of the final version of this article.

## Footnotes

1
http://www.selfcompassion.net/

2
www.wjx.com
